# Can the use of iron phthalocyanine-derivative mouthrinses in COVID-19 patients provide systemic benefits? Research into this potential should be considered

**DOI:** 10.3205/dgkh000500

**Published:** 2024-10-02

**Authors:** Bernardo da Fonseca Orcina, Laura Bertin, Emilene Cristine Izu Nakamura Pietro, Juliana Pescinelli Garcia Kuroda, Lucas Marques da Costa Alves, Fabiano Vieira Vilhena, Paulo Sérgio da Silva Santos

**Affiliations:** 1 Department of Surgery, Stomatology, Pathology and Radiology, Bauru School of Dentistry, University of São Paulo, Bauru, Brazil; 2 Hospital Estadual de Bauru, Bauru, Brazil; 3 TRIALS – Oral Health & Technologies, Bauru,Brazil

**Keywords:** SARS-CoV-2, hospitalization, dentistry, mouthwashes, oral sprays, supporting COVID-19 therapy

## Abstract

**Aim:**

The purpose of this brief report is to discuss the impact of an oral rinse and spray containing an iron phthalocyanine derivative as an additional therapy in hospitalized COVID-19 patients.

**Methods:**

In the first study by this group of authors published on this topic, the clinical status of 22 patients with COVID-19 who were hospitalized and receiving PDMS (phthalocyanine derivative mouth spray) was assessed using the Karnofsky scale (KS) for thtree days (D0, D2, and D4). In another study, the laboratory data (CBC, D-dimer, Ferritin, and C-reactive protein [CRP]) of 41 patients hospitalized with COVID-19 who took part in a randomized clinical trial with an MIPD (mouthwash with iron phthalocyanine derivative) were evaluated retrospectively on the first day of intervention (D1) and 48 hours later (D2). The present study used these data to determine a correlation between clinical symptoms and laboratory data.

**Results:**

In individuals receiving PDMS and evaluated using the KS, a statistically significant intra-group difference (p=0.03, Friedman’s test) was identified. The Durbin-Conover test found a significant difference between D0 and D2 (p=0.008). Laboratory data from only 9 patients in the experimental group and 13 patients in the control group were found in the retrospective analysis. There were no statistically significant confounders in the survival analysis using the Cox regression model. In the descriptive analysis, the intervention group’s CRP was lower than that of the control group.

**Conclusion:**

PDMS demonstrated considerable clinical improvement in patients, whereas MIPD appears to lower CRP, an inflammatory marker, in descriptive analysis.

## Zusammenfassung

**Zielsetzung:**

In diesem Kurzbericht werden die Auswirkungen einer Mundspülung und eines Sprays mit einem Eisenphthalocyanin-Derivat als Zusatztherapie bei hospitalisierten COVID-19-Patienten erörtert.

**Methoden:**

In der ersten Studie wurde der klinische Zustand von 22 Patienten mit COVID-19, die stationär aufgenommen wurden und PDMS (Phthalocyanin-Derivat-Mundspray) erhielten, vier Tage lang (D0, D2 und D4) anhand der Karnofsky-Skala bewertet. In einer weiteren Studie wurden bei 41 Patienten mit COVID-19 im Krankenhaus, die an einer randomisierten klinischen Studie mit einem MIPD (Mundspray mit Eisenphthalocyanin-Derivat) teilnahmen, die Labordaten (CBC, D-Dimer, Ferritin und C-reaktives Protein (CRP)) am ersten Tag der Intervention (D1) und 48 h später (D2) retrospektiv bewertet.

**Ergebnisse:**

Bei den Personen mit KS, die PDMS erhielten, wurde mit Hilfe des Friedman-Tests ein statistischer Unterschied zwischen den Gruppen festgestellt (p=0,03). Der Durbin-Conover-Test ergab einen signifikanten Unterschied zwischen D0 und D2 (p=0,008). In der retrospektiven Analyse wurden nur Daten von 9 Patienten in der Experimentalgruppe und 13 Patienten in der Kontrollgruppe gefunden. Bei der Überlebensanalyse mit dem Cox-Regressionsmodell gab es keine statistisch signifikanten Störfaktoren. In der deskriptiven Analyse war das CRP in der Interventionsgruppe niedriger als in der Kontrollgruppe.

**Schlussfolgerungen:**

Die PDMS zeigte eine erhebliche klinische Verbesserung bei den Patienten, während die MIPD in einer deskriptiven Analyse den Entzündungsmarker CRP zu senken scheint.

## Background

Although the world has been fighting COVID-19 for over three years, much research is still being conducted to better understand its pathophysiology, particularly its late sequelae [1], [2]. Given the evidence for the involvement of the oral cavity in both the pathophysiology and transmission of SARS-CoV-2 [3], [4], [5], [6], [7], [8], our team tested different methods in a number of studies, seeking to evaluate the potential of a mouthwash containing an iron phthalocyanine derivative (MIPD = mouthwash with iron phthalocyanine derivative) as a therapeutic adjunct against COVID-19. An in-vitro study found that a 0.02% concentration of the MIPD rechead a percentage of viral inactivation above SARS-CoV-2 by 99.9% [9]. Furthermore, rinsing and gargling with 5 ml of MIPD for 1 minute 5 times a day yielded improvement in patients with mild/moderate COVID-19 [10] symptoms without the need for hospitalization and without adverse reactions; these symptoms included both oral and upper respiratory tract manifestations of the disease [11], [12]. In another case series, nasopharyngeal and oropharyngeal swabs revealed complete eradication of SARS-CoV-2, P1 variant, within 72 hours of the start of MIPD [13].

In clinical studies with COVID-19 patients undergoing hospitalization, we investigated the phthalocyanine iron derivative in spray and mouthwash form. In the first of several studies [14], we followed 11 patients with mild to moderate symptoms who did not require ICU admission and used the mouth spray (PDMS=phthalocyanine derivative mouth spray). The dosage was three pumps of the spray in the throat, on the tongue and bilaterally on the buccal mucosa, using about 1.5 ml of solution five times a day, which was swished/held in the mouth/gargled for about 30 seconds before the liquid was expelled. Patients were instructed to use this dosage for one week and were monitored by collecting saliva on the first day before intervention, as well as 48 and 96 hours later for RT-PCR analysis. At the end of the follow-up period, 72.8% of the samples tested negative in RT-PCR analysis for SARS-CoV-2 or the patient had been discharged from the hospital [14]. We conducted a triple blind randomized clinical trial (RCT) in this same group of hospitalized patients, but with the MIPD intervention, and found that those in the intervention group had a shorter length of stay (p=0.03) and no need for ICU admission (p=0.02) compared to the control group [15].

Based on these encouraging findings from prior investigations, the purpose of this short communication was to show the impact of topical intraoral interventions on the clinical and laboratory status of COVID-19 patients admitted to the hospital.

## Methods

This controlled trial was conducted in accordance with the principles of the Declaration of Helsinki and ethical standards of human experimentation with the approval of the Human Research Ethics Committee of Bauru School of Dentistry of the University of Sao Paulo, Brazil (CAAE 34,070,620.6.0000.5417). This clinical study was also registered at REBEC–Brazilian Clinical Trial Register (RBR-58ftdj) in 10/28/2020.

This study included patients who used the MIPD or the PDMS, had mild to moderate COVID-19 symptoms, symptom onset within 7 days, did not need to be in the ICU, did not require mechanical ventilation, were between the ages of 18 and 80, and received in-hospital care (World Medical Protocol, antibiotics, corticosteroids, and anticoagulants) in accordance with World Health Organization (WHO) standards [16]. These patients were evaluated to measure the impact of topical intraoral interventions on the clinical and laboratory status of COVID-19 patients admitted to the hospital.

The KS was used to assess the clinical condition of 22 patients who used PDMS. A nurse used the KS to collect data on the first day (D0) of patient assessment, then at 48 hours (D2) and 96 hours (D4). Data normality was confirmed using Jamovi^®^ software (p>0.05). The Friedman and Durbin-Conover ANOVA tests were used to confirm the group relationship, and the Bonferroni correction was used for adjustment. 

Then, to evaluate patients admitted to hospital who had used MIPD, we retrospectively collected the following data from the hospital’s digital records: CBC, D-dimer, ferritin, and C-reactive protein (CRP). Data were gathered from 41 patients who had taken part in a RCT [15]; they were randomly divided into 2 groups: experimental (EG; n=20) and control (CG; n=21). The Cox Regression Model was applied for the survival analysis between the groups using the Jamovi^®^ software. To that end, four covariates and their potential relationships with the need to transfer patients from the clinical ward to the Intensive Care Unit were investigated: intervention (non-active or active mouthwash), patient age, neutrophils, and hemoglobin. 95% confidence intervals (CI) were applied, and a p-value <0.05 was considered significant. 

## Results

**Figure 1:**
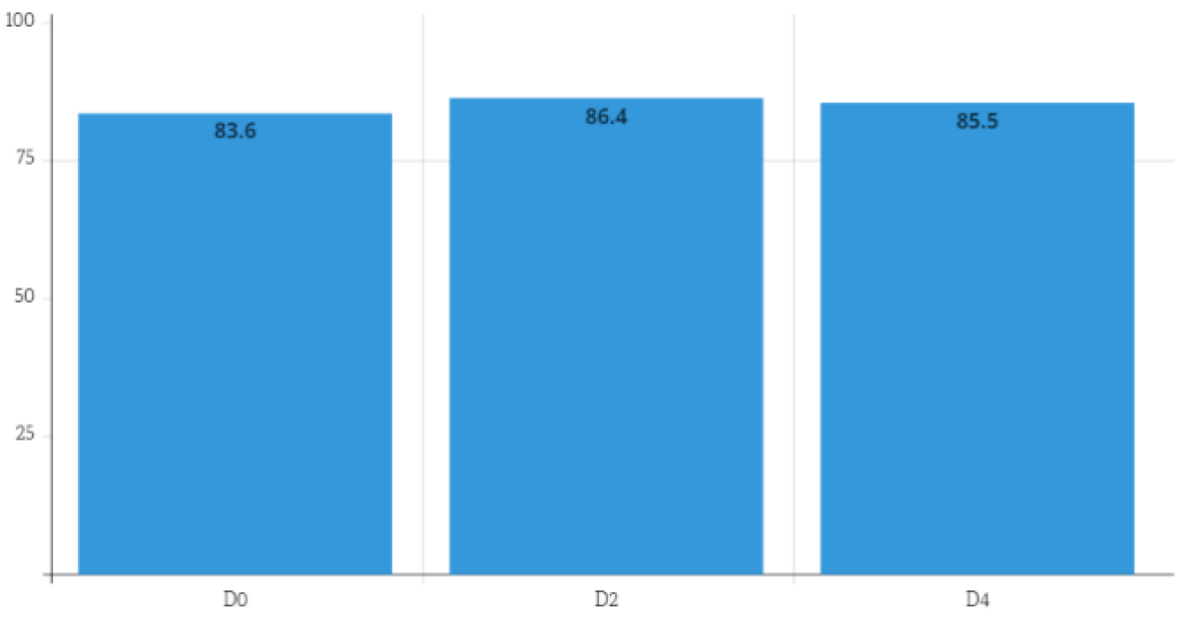
Median values of the Karnofsky scale on each data collection day (D0=first day of clinical parameter collection; D2=48 hours after D0; D4=96 hours after D0).

Intragroup differences were found to be statistically significantly different (p=0.03, Friedman’s test). Only between D0 and D2 (p=0.008) did the Durbin-Conover test reveal a significant difference between the three verified timepoints of the scale (Figure 1 (Fig. 1)).

After searching the hospital system, patients were chosen on the first day of the oral cavity evaluation (D1) and 48 hours (D2) after the MIPD intervention. Only 22 patients in the RCT had laboratory values for D1 and D2: 7 men and 2 women from the EG with a mean age of 60.4 years, and 7 men and 6 women from the CG with a mean age of 50.2 years. Finally, we calculated the mean values of each laboratory parameter in each group of patients at both collection times and performed a descriptive analysis of the results (Table 1 (Tab. 1)). According to the Cox analysis, none of the covariates were related to the outcome of ICU admission (p>0.05). The descriptive analysis showed that only the mean neutrophil values in the EG were lower on D1 than in the CG. On D2, the EG had lower mean values of hematocrit, neutrophils, platelets, and CRP than did the CG. Analyzing the mean values of hemoglobin, hematocrit, neutrophils, typical lymphocytes, platelets, and D-Dimer in the EG and GC on D1 and D2, it was discovered that these were within the mean reference values of the previously mentioned laboratory parameters. Only the CRP showed mean values greater than the reference, in both groups at the two evaluated moments, with the average values having decreased on D2 in the EG and increased in the CG.

## Discussion

### Method

The KS is a clinical measure of a patient’s overall well-being that is widely used in the health sciences, particularly among cancer patients. Its score ranges from 0 to 100 and is divided into tens, with 100 representing a healthy individual and 0 representing a deceased individual [17].

### Results

Due to the lack of studies in the literature that correlate the use of mouthrinses to laboratory parameters of COVID-19 patients or the use of mouth spray and clinical condition measurement through a scale established in the health services in this same patient profile, an study that would perform such an analysis was required. To our research team’s best knowledge, this is the first study to examine these correlations.

Many studies have already demonstrated the antimicrobial and anti-inflammatory properties of phthalocyanines in photodynamic therapies [18], [19], [20], [21], but in the case of MIPD and PDMS, the previously mentioned antiviral properties occurred without photoactivation of the molecule, that is, in the dark. A virtual search in the DrugBank database (https:/www.drugbank.ca/) on a set of over 8,770 FDA drugs based on molecular dynamics simulations and interaction free energies identified phthalocyanine, hypericin, TMC-647055, and quarfloxin derivatives as potential most effective drugs for the treatment of COVID-19. The action of these drugs is signaled by their high affinity for the inner cavity of the spike glycoprotein in the pre-fusion conformation, blocking the HR1 region and preventing SARS-CoV-2 from entering target cells. The main interaction responsible for this inhibition effect in phthalocyanine is hydrophobic. Since phthalocyanines have a similar size and carboxylic acid groups on the periphery that are prone to hydrogen bonding interactions, we expect a higher interaction and affinity of this molecule with the inner cavity in the spike glycoprotein in the pre-fusion conformation. Furthermore, the molecule used in our study is well-known for its ability to interact with and activate oxygen molecules in the air, resulting in highly localized production of activated oxygen molecules. This reactive oxygen has the potential to cause oxidative stress/damage in microorganisms such as SARS-CoV-2, resulting in their inactivation. This hypothesis is supported by the effect of low non-cytotoxic concentrations (1.0 mg/mL to 0.0156 mg/mL) on active viral load reduction. Thus, the high efficacy of the phthalocyanine molecule used in MIPD and PDMS is most likely explained by a dual mode of action, such as blocking the HR1 region and promoting oxidative damage, which leads to virus inactivation [15].

Finally, some of the study’s limitations should be mentioned. First, a high percentage of medical records had incomplete or missing laboratory markers. This is due to the bact that laboratory test requests from patients in the wards vary based on need and clinical condition; there is no routine collection. In addition, the clinical picture of COVID-19 was resolved in many patients, who were then discharged from the hospital. Furthermore, the study only included a small number of people, all of whom were admitted to a hospital unit with mild to moderate conditions, limiting the generalizability of the findings. It is important to remember that conducting clinical research during the pandemic’s critical period hampered sample size calculation, which explains the convenience sample. More robust studies with a larger sample size, a control group, and a longer follow-up period are recommended.

As mentioned above, this is presumably the first study to correlate mouthrinses to laboratory parameters of COVID-19. Despite the fact that the two groups of patients were subjected to the same medical treatment protocol at the time and the mouthwash was an adjuvant therapy, the descriptive analysis of the inflammation markers suggests that the use of the MIPD may have helped reduce systemic inflammation. Furthermore, despite the lack of a control group, the patients who used PDMS showed statistically significanct clinical improvement when the paired analysis was performed.

## Notes

### Competing interests

Dr. F. V. Vilhena has a patent. The other authors declare that they have no conflict of interest.

### Funding

This research was funded by TRIALS–Oral Health & Technologies. The funder contributed to the scope and design of this study; however, they did not influence the collation,management, analysis, and interpretation of the data; preparation, review, or approval of the manuscript; or the decision to submit the manuscript for publication.

### Acknowledgments

This study was financed in part by the Coordenação de Aperfeiçoamento de Pessoal de Nível Superior (CAPES), Brazil (Finance Code 001).

### Authors’ ORCID

- da Fonseca Orcina B: 0000-0003-3367-483X
- Bertin L: 0000-0002-9230-9536
- Izu Nakamura Pietro EC: 0000-0002-4113-3980
- Pescinelli Garcia Kuroda J: 0000-0002-6068-1050
- Marques da Costa Alves L: 0000-0001-9018-6395
- Vieira Vilhena F: 0000-0003-3840-3633
- da Silva Santos PS: 0000-0002-0674-3759

## Figures and Tables

**Table 1:**
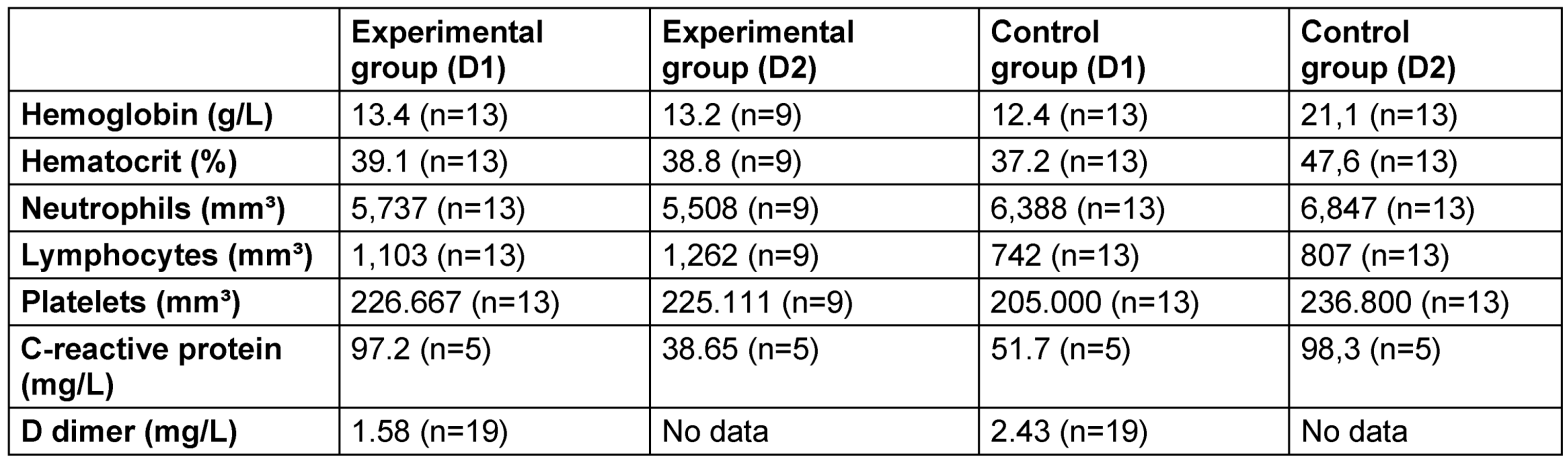
Mean values of each marker in the experimental and control groups on the first day of dental evaluation (D1) and 48 hours (D2) after the MIPD intervention.
